# An Adaptable, Portable Microarray Reader for Biodetection

**DOI:** 10.3390/s90402524

**Published:** 2009-04-14

**Authors:** Deanna L. Thompson, Francesca Pearson, Cynthia Thomas, Rupa Rao, Dennis Matthews, Joanna S. Albala, Sebastian Wachsmann-Hogiu, Matthew A. Coleman

**Affiliations:** 1 NSF – Center for Biophotonics Science and Technology, University of California Davis / 2700 Stockton Blvd. Sacramento CA 95817, USA; E-Mails: dlthompson@ucdavis.edu (D.-L.T.); rupa.rao@abbott.com (R.R.); jalbala@pacific.edu (J.-S.A.);; 2 Lawrence Livermore National Laboratory / 7000 East Ave., Livermore, CA 94550, USA; E-Mails: francesca.pearson@microchipbiotech.com (F.P.); thomas5@llnl.gov (C.T.);; 3 University of California, Davis Medical Center, Department of Neurological Surgery / 860 Y St., Suite 3740 | Sacramento, CA 95817, USA; E-Mail: dlmatthews@ucdavis.edu (D.-L.M.);; 4 University of California, Davis Medical Center, Department of Pathology and Laboratory Medicine / 4400 V Street | Sacramento, CA 95817, USA; 5 University of California, Davis Medical Center, Department of Radiation Oncology / 4501 X Street, Suite G-140 | Sacramento, CA 95817, USA

**Keywords:** Microarrays, immunoassays, protein arrays, point-of-care diagnostics, portable microarray reader

## Abstract

We have developed an inexpensive portable microarray reader that can be applied to standard microscope slide-based arrays and other array formats printed on chemically modified surfaces. Measuring only 19 cm in length, the imaging device is portable and may be applicable to both triage and clinical settings. For multiplexing and adaptability purposes, it can be modified to work with multiple excitation/emission wavelengths. Our device is shown to be comparable to a commercial laser scanner when detecting both streptavidin-biotin and antibody interactions. This paper presents the development and characterization of a handheld microarray imager and directly compares it with a commercial scanner.

## 1. Introduction

Antibody-based immunoassays are currently the most commonly used tools to measure biomarkers in patient samples [1,2]. Since their first use in the 1950s, newer and more efficient antibodies and other affinity reagents have been created to improve assay sensitivity. The development of antibody microarrays has played an increasingly important role in biology and medicine [3–5], and tremendous progress has been made in the development of new array-based technology platforms for biological research. Microarrays offer several distinct advantages over conventional analytical technologies, including requiring only small sample and reagent volumes, and offering high-throughput parallel analysis [4,6–10]. This technology has been used successfully to detect and quantify specific target proteins in complex mixtures such as clinical samples [6,11].

New diagnostic tests, such as microarray-based assays for breast cancer [11] or for autoimmune diseases [12], have the potential to dramatically change the face of medicine. However, the mainstream medical community is often slow to adopt new technologies. Some of the reluctance is due to the cost of new technology, unwieldy equipment unsuitable for an office setting, or lack of time to learn to use a complicated system. There is, therefore, an increasing demand for simple, point-of-care (POC) diagnostic assays and readers capable of providing rapid, sensitive and quantitative results.

POC integrated systems possess the ability to process clinical samples for a number of different types of biomarkers in a variety of settings, such as clinical laboratories, patients’ bedsides, and doctors’ offices. Automation of clinical diagnostic tests is also highly desirable as it would improve the efficiency of the laboratory and reproducibility of the testing procedure by eliminating human error [13]. This is especially necessary in situations where assays need to be performed without the use of a laboratory or trained individuals. In addition, implementation of POC technologies should be, by their very nature, low cost.

Microarray assays can be adapted to fit fully on a standard microscope slide for POC applications. After a brief incubation period, the only equipment needed to analyze the results of a preprinted assay is a microarray reader and a simple image processor. However, current microarray laser scanners/readers cost tens of thousands of dollars, are space prohibitive, and require a trained operator. Ideally a small, inexpensive, and specialized reader would complement the slide-based assay.

One of the challenges in creating such a reader is miniaturizing the light source without drastically reducing the excitation light. Bavykin *et al.* previously developed a portable microarray imager [14] that utilizes a small laser diode, but their use of a film-based camera precluded rapid analysis of the array. Another group later demonstrated a digital portable microarray reader [15] using an ultra-bright LED for excitation, but their system was designed for laboratory use rather than as a clinical prototype. Although both systems were smaller than traditional scanners, they cannot be easily stored and handled in doctors’ offices, and neither group provided comparative validation of their prototypes using a commercial scanner. Vo-Dinh *et al*. [16] developed a small biosensor platform that overcomes some of these barriers, but lacks the ability to easily adapt to new sizes or shapes of fluorescent patterns.

In this paper we describe a new alternative to microarray scanners that would meet the needs of the market. By addressing some of the drawbacks of commercial scanners, such as size, cost, and complexity, our specialized device will appeal to those looking for POC microarray solutions. While the handheld microarray reader described here lacks some of the features and functions of the larger laser scanners, its small and minimalistic design makes it ideal for specialized uses such as rapid diagnosis in medical clinics and other POC settings. Additionally, when combined with a lateral flow assay such as previously described [11], the sample does not need to be sent to a separate laboratory for processing. Although our reader does not scan a full slide, we believe that our field of view is sufficient to accommodate the typical number of biomarkers to be used for a single POC assay, thus easily fitting within one image. Furthermore, in recognition that accuracy is of the utmost importance for acceptance by medical providers, we provide here a direct comparison of our handheld imager to a commercial scanner.

## 2. Results and Discussion

### 2.1. Design and characteristics of the handheld microarray imager

**Figure 1.**
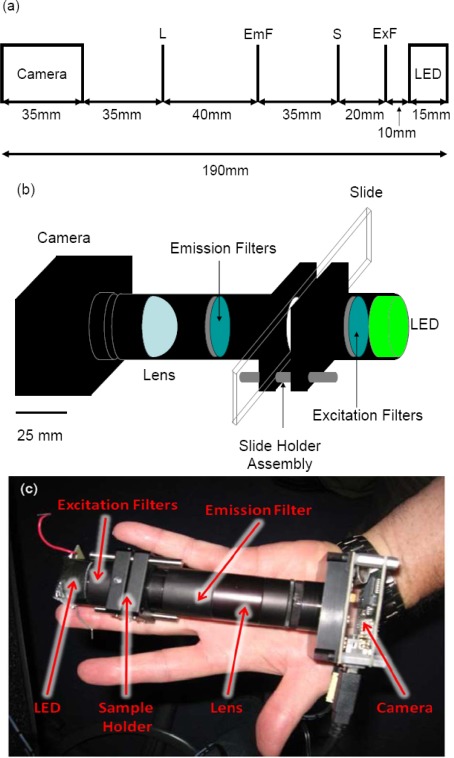
Diagrams and picture of the actual device. All measurements are in millimeters (a) Optical layout of the device. L = lens with a 25.4 mm focal distance, EmF = emission filters, S = sample, ExF = Excitation filters. (b) Schematic of hand-held microarray reader. (c) Actual image of the hand-held reader.

The design of the microarray reader presented here combines simplicity with multifunctionality. Figure 1 shows the optical layout, a 3D schematic, and a photograph of our prototype. Measuring only 19 cm in length with a base of 6.5 cm × 5 cm, our imager is small enough to be handheld. Built from easily obtainable materials for under $3,000.00, it contains no moving parts, so its durability is limited only by the ruggedness of the CCD detector. A high-powered LED provides the excitation light, which is narrowed by a set of filters before illuminating the field of view with approximately 4 mW of 530 nm light. This translates to roughly 16 × 10^15^ photons per second per square centimeter, or 3 × 10^12^ photons per second per microarray spot. The light propagating in the forward direction then passes through another set of filters so that only the fluorescent light from the microarray is focused onto the detector. The high inherent numerical aperture (NA = 0.45) of the focusing lens allows for high collection efficiency. From the CCD, a 16-bit fluorescence image is captured and saved (via USB connection to a laptop computer) for image analysis and processing. With a field of view of approximately 7.5 × 10 mm, a 30 × 40 array of spots spaced by 250 μm spot-to-spot both horizontally and vertically could conceivably be visualized in one image by this device.

The forward detection is preferable to alternatives using dichroic mirrors for several reasons including cost, simplicity, and size. One specific benefit of using forward detection is the ability to easily change wavelengths. All of the filters and the LED are easily accessible, and exchanging the filters and LED does not affect the alignment (as would be the case with a dichroic mirror). This allows the device to be used for multiple applications, with different wavelengths beyond our initial experiments where Cy3 and Cy5 dyes were used.

**Figure 2.**
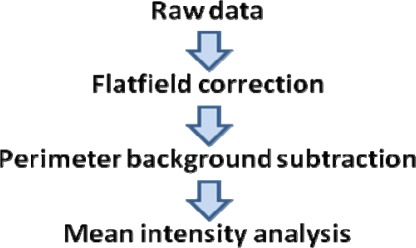
A flowchart depicting how a microarray image is processed.

Computer-based algorithms were implemented to turn the imaging system into a reader, shown as a flowchart in Figure 2. The first process corrected for uneven light distribution from the LED. This simple step was applied to each image and verified by comparing two images recorded at different angles. Next, mean intensities of the spots and their perimeters were gathered, and background subtraction using this information was performed. These steps yielded the corrected mean spot intensities used for analysis, which were found to be comparable to the values obtained from a commercial laser scanner. These processes are described in detail in the Experimental section.

Additional upgrades are possible to improve portability. First, a program automating all of the steps described above is needed, such as provided by the *UCSF Spot* program [17]. With arrays in a fixed position relative to the detector, the same pixel numbers can be used every time to gather means and SDs, or object-recognition software can be used to select spot borders. The next optimization step is to reduce the number of separate wires required. Currently the LED is powered by a variable power supply, but it has also been successfully run off a pair of C batteries. Future designs could power both the LED and the camera (which currently requires a standard electrical outlet) through a USB connection. This would allow the device to be taken to remote areas, requiring only a standard laptop to both power the device and process the images. The programs required to analyze the image could all be included on a chip, along with application-specific programs, and wired directly into the device. This would transition our handheld microarray imager into a fully contained, highly specialized handheld microarray reader. Furthermore, the cost of the reader can be reduced much below the current $3,000 figure. By using commercial rather than research products, components such as the optical tube or the camera can be acquired at a much lower cost.

### 2.2. Comparative Analysis

**Figure 3.**
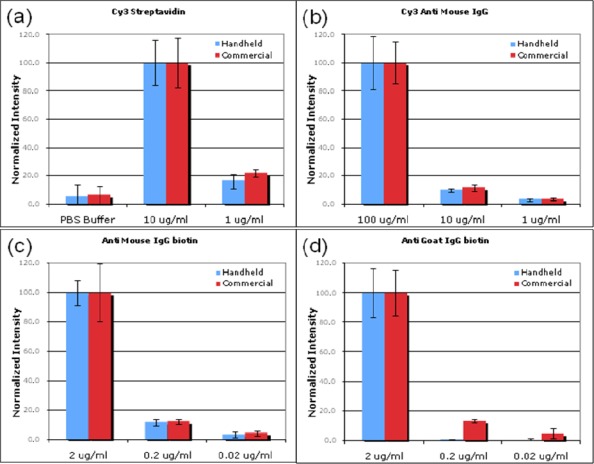
Comparative analysis of the handheld reader’s results with those of a traditional scanner. Each graph was normalized so the brightest spots each had intensity values of 100. Error bars represent a total of two SDs for each spotting group. (a) Intensities of the PBS spots and the 10 : 1 printing ratio of Cy3 streptavidin are plotted. (b,c) The 100 : 10 : 1 printing ratios are reflected in the fluorescence intensity ratios for both of these spotting groups. (d) The handheld scanner reaches its detection limit for the concentrations shown of anti goat IgG biotin where the SD is larger than the mean intensity value.

**Figure 4.**
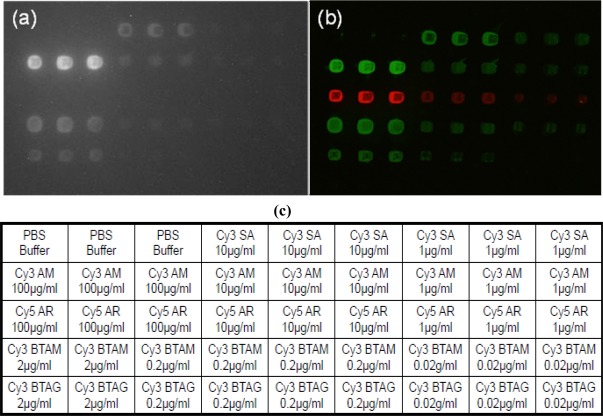
Scanned images. (a) A scanned image taken with the hand-held imager. (b) A composite false-color image from the GenePix 4000B scanner of the same spotted array. The green spots in (b) are labeled with Cy3, whereas the red spots are labeled with Cy5, which is undetectable using the current filter setup of the handheld imager. (c) A legend of the protein and concentration used for each array spot. They layout of the chart matches the layout of the spots in the arrays shown. B = PBS buffer, SA = streptavidin, AM = anti mouse, AR = anti rabbit, BTAM = anti mouse biotin, BTAG = anti goat biotin. The dye is spotted directly onto the array for the first three rows only. A later incubation step conjugates the dye to the biotin for the bottom two rows.

For each antibody or protein type, the intensities collected from both a traditional laser scanner and the handheld device were normalized so that the brightest concentration on each had a value of 100. Figure 3 shows the normalized comparison of the intensities and their SDs from the two devices, and Figure 4 shows the images of the microarrays and a corresponding spot-array layout. The antibodies were each printed in concentration ratios of 100:10:1, with the exception of the Cy3 streptavidin, which was printed only at the lower two concentrations. The first three spots contain only PBS buffer. The intensity plots in Figures 3b, c clearly correlate with the expected spotting ratios. The detection limit of the reader for low concentrations with a 40 s exposure time becomes evident in Figure 3d, where the SD of the anti goat IgG biotin 0.02 μg/mL spots (SD = 78) is larger than the mean intensity value (I = 51) after background subtraction.

The SDs for the handheld reader, as a percentage of the correlated mean intensities, ranged from 9% to 183%, whereas the commercial scanner ranged from 10% to 116%. Looking at groups of spots with 3 orders of magnitude of detectable intensities, specifically the spots represented in Figure 3b,c, the SDs were greatly reduced. For the handheld scanner, this narrows to 9% to 55% with a mean of 25% and the highest percentages at the weakest spots. For the scanner, the range is 14% to 40%, with a mean SD of 22%.

These results suggest that this microarray reader is an excellent replacement for several applications requiring multiple order-of-magnitude intensity detection. For a single exposure time, our device is able to detect intensities spanning three orders of magnitude, which is comparable to the commercial scanner used. By taking multiple images with different exposure times, the dynamic range can be further extended. For example, by taking three exposures at 10 ms, 1 s, and 100 s, a total of seven to nine orders of magnitude of intensity could reasonably be covered. This would require that spots be spaced far enough apart to avoid the overlap of saturated CCD pixels corresponding to bright spots with pixels corresponding to adjacent spots, which is easily accomplished with the large field of view of our device. For example, doubling the spot-to-spot distance to 500 μm would still allow detection of an array of 15 × 20 spots within a single image.

Another important feature of microarray readers is the ability to detect the difference between two intensities that are within the same order of magnitude. For these types of applications where low SDs are important, multiple images with longer exposures are needed to improve signal-to-noise ratio. As mentioned above, brighter spots (close to the saturation value of the camera) were observed to have SDs that were relatively low compared to the mean intensity.

## 3. Experimental Section

### 3.1. Handheld microarray imager

#### 3.1.1. Imager structure

A Thor Labs 25.4 mm optical tube encloses the entire device, with the exception of the CCD camera, thus blocking outside light from interfering with fluorescence detection. The slide holder is a Thor Labs cage assembly, with two rods supporting the slide from below and the cage assembly plates sandwiching the slide to hold it in place.

#### 3.1.2. Light source

At one end of the tube, an ultra-bright Luxeon® Star with Optics LED, powered at 3.3 V, provides illumination to back illuminate a standard microscope slide. The light emitted has a peak wavelength of 530 nm with a range of 520 – 550 nm, and the power was measured to be approximately 80 mW.

#### 3.1.3. Filters

A set of two 532/10 nm filters (Chroma Technology Corporation, VT, USA) narrows the excitation wavelength range to 527 – 537 nm. The excitation light and the fluorescence signal coming from the sample slide passes through three more filters. The first two filters, a 572 nm longpass (Chroma) and a 590 nm longpass (Andover Corporation, NH, USA), block the excitation wavelengths. The third filter, a 592/100 nm bandpass filter (Chroma), stops the infrared signature from the LED from reaching the camera.

#### 3.1.4. Lens

The tube lens used has a 25.4 mm focal length and diameter from Thor Labs. The inherent NA is 0.45, while the NA for our collection geometry is 0.17. Because the light from the sample is not collimated, the distance between the sample and the lens is much greater than the focal distance, resulting in the lower NA.

#### 3.1.5. Camera

The camera, an LU130M (Lumenera Corporation, Ottawa, Canada), has a ½” monochromatic chip with a pixel size of 4.65 square μm and a 60 dB dynamic range. The manufacturer lists a dark count of 2 electrons (per pixel per second) at 25 °C with a readout noise of 8 electrons, and a quantum efficiency of 40% at 500 nm.

### 3.2. Microarray manufacturing

#### 3.2.1. Comparative analysis slides

Reagents were spotted at concentrations shown in Figure 4. PBS was printed as a negative control. One labeled peptide and four different antibodies were printed as positive controls: Cy3 streptavidin (Cy3 SA), Cy3 anti mouse IgG (Cy3 AM), anti mouse IgG biotin (BTAM), anti goat IgG biotin (BTAG) (Rockland Immunochemicals, Inc. Gilbertsville, PA, USA), and Cy5 anti rabbit IgG (Cy5 AR) (Zymed Invitrogen, Carlsbad, CA, USA). The antibodies were diluted in 2x Protein Arraying Buffer (Schleicher & Schuell, Whatman, Kent, UK) to a total volume of 15 μL in a 96-well titer plate. Approximately 10 nL of each solution was printed on Corning GAPS II Slides (Invitrogen, Carlsbad, CA, USA) using the OmniGrid Accent (Genomic Solutions, Ann Arbor, MI, USA) arrayer. Slides were crosslinked for 5 mins using UV irradiation. Four subarrays were printed on each slide, and the spacing was 250 μm spot-to-spot both horizontally and vertically within each subarray. The array layout can be seen in Figure 4c. Prior to use, the slides were blocked for 1 hour using PBS containing 1 mg/mL of BSA. The slides were then washed three times in 1X PBS containing Tween^®^ 20. For detection, a total of 800 μL of Cy3 SA in PBS was added to the array. The Cy3 SA interacts with the biotin to allow detection. After a 10 minute incubation period, slides were again washed three times for five minutes in 1X PBS before air drying.

#### 3.2.2. Flatfield correction slide

The flatfield correction slide was prepared in a manner similar to that of the comparative analysis slides. ATM synthetic peptide was used, and 10 × 10 spot subarrays were printed in a 2 column, 4 row format. Spacing of spots in both the vertical and horizontal position is 300 microns, except for the first array which has spacing of 500 vertically and 300 horizontally.

### 3.3. Flatfield Correction:

**Figure 5.**
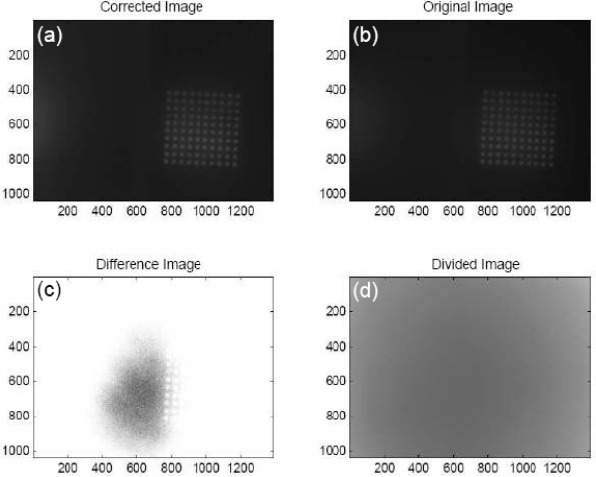
Results of the flatfield correction program. (a) The image as it appears after being corrected. (b) The original image. (c) The adjusted image subtracted from the original image. (d) The adjusted image divided by the original image.

**Figure 6.**
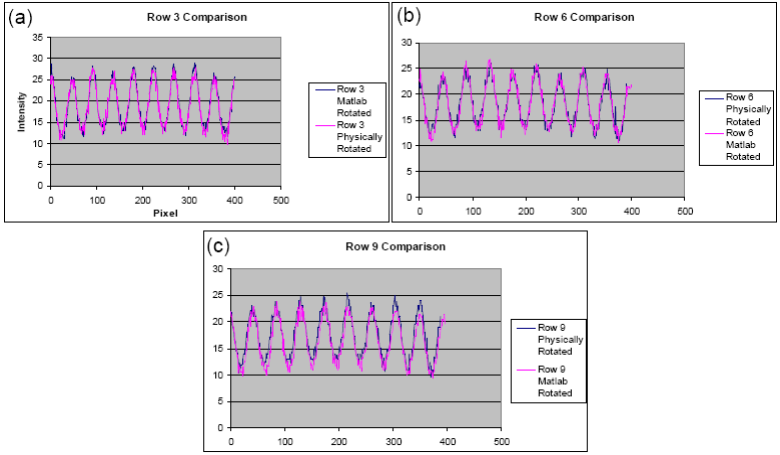
Comparisons of row histograms of two images of the same array. Blue lines represent physically rotated arrays and pink lines represent computer rotated arrays. The agreement between the two histograms indicates a successful flatfield correction program.

Each image was flatfield corrected using one of two equivalent methods. The first was to convert the images into MATLAB matrices using the ‘imread’ function in the Image Processing Toolbox. Each matrix entry *i, j* from the original image was divided by the corresponding entry *i, j* from the fluorescent slide’s matrix. The new matrix was then scaled to the same mean value as the original image’s matrix. Figure 5 shows a comparison of an original and corrected image, including the divided difference and subtracted difference. The final matrix was converted into a bitmap image and saved as a new file. Alternatively, the flatfield correction could be done using Image J. The original image was divided by the calibration image using the ‘Image Calculator’ function. The new image was then multiplied by the mean intensity of the calibration image, returning it to the same mean intensity as the original image. To test this method, two images were taken of the same array. The slide was physically rotated 90° between the two pictures, and a third image was taken of the calibration slide. Each of the two array images was flatfield corrected using the MATLAB program. The second image was rotated to match the orientation of the first, and a defect in the array was used to confirm the match. The ‘Plot Profile’ feature of ImageJ measured the intensity along rows in the microarray. The data was extracted to Microsoft Excel, and the intensities were normalized to a mean value of one. The normalized intensities of one row on the first image were plotted versus the pixel number, and the same data from the second image was superimposed on it (Figure 6). Three of ten rows were analyzed in this manner.

The comparison between the physically rotated and the computer rotated images showed the peaks in each row matching in both height and position. Some discrepancy was noticed in the mean intensity between the two images, but the difference in each row was only 1 – 4%. This can be attributed to differences in ambient light during the experiment combined with human error in selecting the line to be evaluated using ImageJ. Changes in background counts were observed after the flatfield correction, but this was expected since the calibration takes into account the entire image, not just the array.

### 3.4. Background Subtraction

To correct for the background caused by scattered excitation light, room light, or detector noise, the background around the perimeter of each spot was calculated and subtracted. First, the mean intensity of a large circle around each spot was gathered using ImageJ. Next, the mean intensity of a smaller, inscribed circle approximately enclosing the spot was measured. The average intensity of the outer ring, not including the inner circle, was calculated:

(1) $${\mathrm{Mean}}_{\mathrm{donut}}=\frac{{\mathrm{Mean}}_{\mathrm{big}}*n_{\mathrm{big}}-{\mathrm{Mean}}_{\mathrm{small}}*n_{\mathrm{small}}}{n_{\mathrm{big}}-n_{\mathrm{small}}}$$

where *n* represents the number of pixels in each area, *big* indicates the circle with larger radius, and *small* indicates the circle with the smaller radius. This yielded the mean background around each spot, approximately 16,000 – 29,000 counts for a 40 s exposure. After calculating it for each spot, the mean background was subtracted from the mean intensity of the smaller circle. This technique provided a locally accurate background subtraction, rather than a less accurate bulk background subtraction method. Corrected mean intensities then ranged from approximately −300 (rare) to greater than 10,000 for the comparative analysis slides. The flatfield correction slides were not background subtracted.

### 3.5. Comparative Protein Array Analysis

Using the handheld microarray reader, images with 40 second exposure times were recorded of each of the total of 8 arrays printed on the two slides described in Section 3.2. The exposure time was chosen to avoid saturation of the camera and also to capture a range of intensities spanning several orders of magnitude. Images were then flatfield corrected and background subtracted.

Image analysis was performed using ImageJ. A circle of fixed radius was hand-centered on each spot in the arrays, with the radius chosen to encompass the largest spot. The mean intensities and SDs of each spot were measured, then recorded and analyzed in an Excel spreadsheet.

A GenePix 4,000 B commercial scanner (Axon Instruments, Union City, CA, USA) was used to take scans of the same slides. The PMT gain was set to 400 for Cy3 (532 nm) and 600 for Cy5 (635 nm). The included software provided the mean intensities and SDs used in this analysis.

With each antibody mixture spotted in triplicate per array, this yielded a total of 18 spots per antibody type and concentration to be analyzed. The SDs shown in our results (the error bars in Figure 3) were calculated from these 18 data points. Only three out of four arrays per slide were analyzed.

## 4. Conclusions

We have developed a relatively low-cost portable reader capable of detecting intensities over multiple orders of magnitude. Although other handheld imagers have been developed [14], to our knowledge no direct comparison with traditional microarray scanners has been previously reported. With our device, the mean SD in intensity detection was only 25%, compared to 22% for a commercial laser scanner. This high degree of comparability makes it a reliable choice for POC testing. Also amenable to a POC setting, the device is small enough to be handheld, measuring only 19cm in length. It has the potential to be powered and run entirely by a laptop or to be self-contained with a software chip and battery. In addition the device could be quickly adapted for a variety of assay formats, including lateral flow assays.

Because it is powered by an LED rather than a laser, and the filters are easily accessible, our reader also is flexible in its excitation and emission spectra. The ability to use a variety of dyes makes our reader applicable to both the clinical and laboratory setting, and highly adaptable for many microarray-based tests. Also unlike previous handheld imagers, the digital format of our imager allows the information to be directly recorded on a computer or other electronic device for quick analysis. In the future our device could potentially send a diagnosis directly to a doctor’s or technician’s PDA, dramatically increasing the ability to treat a patient without delay.
